# Peripheral clearance of brain-derived Aβ in Alzheimer's disease: pathophysiology and therapeutic perspectives

**DOI:** 10.1186/s40035-020-00195-1

**Published:** 2020-05-07

**Authors:** Yuan Cheng, Ding-Yuan Tian, Yan-Jiang Wang

**Affiliations:** 1 Department of Neurology and Centre for Clinical Neuroscience, Daping Hospital, Third Military Medical University, Chongqing, China; 2 The Institute of Brain and Intelligence, Third Military Medical University, Chongqing, China; 3 Key Laboratory of Aging and Brain Diseases, Chongqing, China; 4 Center for Excellence in Brain Science and Intelligence Technology, Chinese Academy of Sciences, Shanghai, China

**Keywords:** Alzheimer’s disease, Beta-amyloid (Aβ), Blood-brain barrier, Lymphatic vessel, Venous sinus, Periphery, Liver, Kidney, Intestine, Skin, Blood, Monocyte, Enzymes

## Abstract

Alzheimer’s disease (AD) is the most common type of dementia, and no disease-modifying treatments are available to halt or slow its progression. Amyloid-beta (Aβ) is suggested to play a pivotal role in the pathogenesis of AD, and clearance of Aβ from the brain becomes a main therapeutic strategy for AD. Recent studies found that Aβ clearance in the periphery contributes substantially to reducing Aβ accumulation in the brain. Therefore, understanding the mechanism of how Aβ is cleared in the periphery is important for the development of effective therapies for AD. In this review, we summarized recent findings on the mechanisms of Aβ efflux from the brain to the periphery and discuss where and how the brain-derived Aβ is cleared in the periphery. Based on these findings, we propose future strategies to enhance peripheral Aβ clearance for the prevention and treatment of AD. This review provides a novel perspective to understand the pathogenesis of AD and develop interventions for this disease from a systemic approach.

## Background

Alzheimer’s disease (AD) is the most common form of dementia, and disease-modifying therapies are not available to date. The extracellular senile plaques formed by the deposition of amyloid-beta (Aβ) peptide are the specific hallmark of AD. Aβ is generated from sequential cleavages of the amyloid precursor protein (APP) by BACE-1 and the γ-secretase complex. Compelling evidence supports the pivotal role of Aβ in the pathogenesis of AD. This evidence includes the following: (1) overproduction of Aβ in the brain owing to mutations of APP or presenilin genes 1/2 (PS1/2) in familial AD and an additional copy of the APP gene in Down syndrome clearly causes AD-like dementia [1]. (2) Reduction of Aβ production due to a missense mutation (p.A673T) in the APP gene results in a reduced risk for AD in an Icelandic elderly population [2]. (3) The apolipoprotein E (ApoE) ε4 allele, the strongest genetic risk factor for AD, is closely involved in the regulation of Aβ metabolism [3]. (4) In the trajectory of AD, abnormal Aβ accumulation precedes neurodegeneration and cognitive decline in both familial AD and sporadic AD [4, 5]. This evidence suggests that the abnormal metabolism of Aβ in the brain plays a central role in the pathogenesis of AD.

Converging data from animal models and clinical studies have demonstrated that abnormal Aβ accumulation in the brain causes neurodegeneration, neuroinflammation, impaired neuronal function, and ultimately cognitive decline. This process is mainly caused by the overproduction of Aβ due to mutations in the APP and PS1/2 genes in familial AD, which accounts for 1% of total AD patients, while dysfunction of Aβ clearance is hypothesized to be the main reason for Aβ accumulation in sporadic AD, which accounts for 99% of total AD patients [6]. Therefore, improving Aβ clearance has become a promising therapeutic strategy for AD [7]. Indeed, several potential pathways have been shown to be involved in Aβ clearance from the brain, including phagocytosis and endocytosis by various cells, such as microglia, perivascular macrophages and astrocytes, and proteolytic degradation by enzymes, including neprilysin (NEP), insulin-degrading enzyme (IDE) and matrix metalloproteinases (MMP). Recent studies have shown that high levels of Aβ could flow from the brain to the periphery, and physiological catabolism of brain-derived Aβ in the peripheral system has been revealed in both humans and mice [8], providing a novel perspective for understanding the pathogenesis of and developing therapeutics for AD. The aim of this review is to discuss the recent findings on the peripheral clearance of Aβ and its potential for AD prevention and treatment.

## Main text

### Mechanisms of Aβ efflux from the brain to the periphery

Several pathways, including the blood-brain barrier pathway, lymphatic-related pathway and arachnoid granule pathway, have been shown to mediate Aβ effluxes from the brain into the periphery.

#### Blood-brain barrier pathway

Numerous studies have suggested that pathological changes in the neurovascular unit, which includes clusters of glial cells, neurons and pericytes, contribute to the onset and progression of AD and support a link between blood-brain barrier (BBB) dysfunction and neurodegeneration [9]. In addition, the capillary length in the mouse and human brain is approximately 0.6 km and 650 km, respectively, which accounts for > 85% of the total cerebral blood vessel length, providing a large endothelial surface area for substance exchanges between the blood and brain. The BBB is, therefore, considered to be the primary approach to eliminate pathological molecules such as Aβ from the brain [10].

Aβ efflux is normally mediated via its receptors on the brain endothelium, and the clearance mechanism is mainly mediated by cell surface proteins, mainly low density lipoprotein receptor-related protein 1 (LRP1), which localizes predominantly on the abluminal side of the cerebral endothelium [11]. Some LRP1 ligands co-deposit with Aβ in senile plaques and are involved in Aβ clearance, such as ApoE, α2-microglobulin (α2M), and tissue-type plasminogen activator [12]. The affinity of Aβ40 for LRP1 is higher than that of Aβ peptides with a greater β-sheet content, such as Aβ42, leading to Aβ42 peptides being less efficiently cleared from the brain [13]. Furthermore, the LRP1-mediated Aβ clearance across the BBB was shown to be very rapid, with a rate of 0.21 pmol Aβ/minute/g interstitial fluid (ISF) for Aβ40 and 0.11 pmol Aβ/minute/g ISF for Aβ42 [14].

LRP1 works closely with phosphatidylinositol-binding clathrin assembly protein (PICALM) to clear Aβ monomers, oligomers, and aggregates from the brain across the BBB. Another key protein, P-glycoprotein (Pgp, also known as ABCB1), which localizes predominantly on luminal membranes of brain endothelial cells, also mediates the active efflux of Aβ from the brain to blood. Due to its special anatomical location, LRP1 has been suggested to facilitate the initial uptake of Aβ from the ISF, followed by Pgp pumping Aβ out of the endothelial cells into the blood [15, 16]. In addition, LRP2, the largest receptor of the low-density lipoprotein receptor (LDLR) family, facilitates the endocytosis of Aβ as well as its clearance across the BBB after binding to apolipoprotein J (ApoJ, also named clusterin) [13]. In addition to these mechanisms, some other receptors mediate Aβ efflux, such as insulin-sensitive transporters and ANP-sensitive transporters [17]; however, the relative contribution of these receptors remains unclear, and more research is required to elucidate their role in the pathogenesis of AD.

In contrast, circulating Aβ enters the brain mainly through receptor for advanced glycation end products (RAGE), a multiligand influx receptor in the immunoglobulin superfamily that is expressed on the luminal surface of brain vessels [18]. The BBB levels of LRP1, Pgp, and RAGE are changed in AD models before Aβ deposition, which may contribute to Aβ accumulation in the brain [10].

#### Arachnoid granule-venous sinus pathway

The role of the BBB in material exchange and transportation is well known; however, there are still other ways to transport brain-derived metabolites to the periphery. Several lymphatic-related clearance routes and arachnoid granule pathways drain solutes from the brain into the periphery.

Cerebrospinal fluid (CSF) is also directly drained to peripheral blood via arachnoid villi and granulations in the walls of major venous sinuses [19]. The bulk flow of CSF into the blood appears to occur via large vacuoles that form on the abluminal side of the endothelial cells, passage through the cells and release of the contents into the venous blood [20]. The majority of the CSF in humans appears to drain by this route; however, in newborn lambs, the nasal pathway is the primary route, as arachnoid villi do not develop until later in development [21, 22]. In addition, there is considerable species variation in the size and structure of arachnoid villi and granulations; for instance, arachnoid villi of experimental animals are much smaller and simpler than human arachnoid granulations, suggesting that animal studies may not truly reflect human physiological condition. Therefore, the contribution of arachnoid granule-venous sinus pathway in transporting Aβ and other metabolites from the brain to the periphery in human needs to be investigated.

#### Lymphatic-related pathways

The first lymphatic pathway is the meningeal lymphatics at the bottom of the rodent skull, which are specialized to drain CSF to deep cervical lymph nodes, allowing wastes and other macromolecules to leave the brain [23]. However, the contribution of lymphatics in draining Aβ out of the brain is unclear, although disruption of meningeal lymphatics accelerates AD pathologies in the brain of animal models [24]. The meningeal lymphatics have also been revealed in human beings [25], their physiological functions in maintaining the brain homeostasis and their aberrant alterations in the pathogenesis of AD remain largely unknown. But the finding of meningeal lymphatics provides a novel perspective to understand the process of Aβ clearance from the brain and suggests a new intervention approach for AD.

The second lymphatic pathway is the perineural outflow pathways through which CSF drains to deep cervical lymph nodes. Emerging evidence has shown that CSF outflows along the cranial nerves, especially in the nasal or optical regions [26, 27]. More specifically, perineural drainage along the olfactory nerve, the first cranial nerve (CN I) through the cribriform plate to reach the nasal mucosa is considered to be the most important CSF lymphatic outflow route in several species [27–30]. In addition, the optic nerve (CN II) and trigeminal nerve (CN V) exhibit a perineural pattern [27]. All these pathways are potential drainage routes to clear toxic proteins, such Aβ, from the brain.

The third lymphatic pathway is the perivascular pathway which drains ISF from the brain to the cervical lymph nodes through basement membranes in the walls of capillaries, the tunica media of arteries, and the wall of the internal carotid artery in the neck [19]. Aβ in the basement membranes of capillaries and artery walls in cerebral amyloid angiopathy (CAA) almost certainly blocks the perivascular drainage pathways, leading to increasing impedance of solute drainage from the brain. With advancing age, the artery walls become less elastic, and stiffening may interfere with perivascular drainage of ISF and solutes in elderly individuals [31].

The fourth lymphatic pathway is the paravascular space which is a narrow space between the irregular surface of the leptomeningeal and the outer basement membrane of the vessel walls and that of the glia endfeet [32, 33], and it has been proposed to be part of the glymphatic pathway [34]. This cortical paravascular route was referred to as the Virchow-Robin space (VRS); however, the exact boundaries of the VRS are not clearly defined to date. Paravascular drainage of solutes from the CSF appears to be dependent on the expression of aquaporin 4 (AQP4) and on efficient arterial pulsations, suggesting that pulsations in artery walls may generate the motive force for the transport of solutes out of the brain [35, 36].

There appears to be a difference in the drainage pathway between the CSF and ISF, and little is known about the proportion of ISF that drains into the CSF. A previous study indicated that 10–15% of the ISF drains into the CSF in rats [37], and a similar degree of drainage may occur in humans [19]. In addition, another research demonstrated that direct transport of Aβ across the BBB accounts for ∼25% of Aβ clearance, and absorption of Aβ in the CSF accounts for ∼25% of the total CNS Aβ clearance in humans [38]. However, the main mechanism through which Aβ leaves the brain and the exact contributions of each of these pathways to overall Aβ clearance remain unknown. Nevertheless, these pathways work synergistically to drain pathological proteins, such as Aβ, from the brain to the periphery, indicating that the peripheral tissues and organs are physiologically related to the metabolism of brain-derived wastes [39].

### Clearance of brain-derived Aβ in the periphery

It is estimated that Aβ clearance via the BBB is reduced by approximately 30% in AD patients [40]. However, the amount and mechanisms of Aβ clearance in the periphery are poorly understood. Previous studies have suggested that approximately 40%–60% of brain-derived Aβ is cleared in the periphery [8, 41, 42]. A recent study showed that deep cervical lymph node ligation aggravates the AD-like pathology of APP/PS1 mice [43], suggesting that blockage or dysfunction of the brain drainage routes is one of the factors that contribute to AD progression. Targeting meningeal lymphatics with vascular endothelial growth factor C (VEGFC), an essential growth factor for lymphatic endothelial cells, enhances the meningeal lymphatic drainage of CSF macromolecules and improves learning and memory performance in animals [24, 44].

All these findings imply that there are physiological mechanisms to transport pathological molecules from the brain to the periphery for clearance and suggest that peripheral clearance has a crucial role in removing brain-derived Aβ. In the following section, we discuss where and how brain-derived Aβ is cleared in the periphery.

#### Blood component-mediated Aβ clearance

A recent study demonstrated that bone marrow transplantation reversed the age-related impairments in cognitive function and synaptic plasticity in aged mice [45]. Another study showed that bone marrow-derived cells contribute to the recruitment of microglial cells in response to Aβ deposition in APP/PS1 mice [46]. Indeed, multiple components in the blood have been shown to participate in circulating Aβ clearance.

##### Enzymes

Secreted enzymes, which have an affinity for specific domains within the Aβ amino acid sequence and an ability to cleave these peptides to shorter, more benign forms, are critical for the catabolism of circulating Aβ. These proteins include insulin-degrading enzyme (IDE), neprilysin (NEP) and its homologue endothelin-converting enzyme (ECE), angiotensin converting enzyme (ACE), matrix metalloproteinase-9 (MMP-9), and plasmin, the key enzyme of the plasminogen system.

IDE is a well-validated Aβ-degrading enzyme that was originally isolated as a molecule regulating plasma insulin levels. It is mainly a soluble cytoplasmic enzyme, although it also exists in a secreted form [47]. IDE activity levels were inversely correlated with brain Aβ burden [48], and IDE knockout animals showed a significant increase in the brain Aβ levels, suggesting that loss of this activity may contribute to AD pathology [49].

NEP is a ubiquitous circulating protease and is abundant in the kidney and the lung. NEP is considered to be the most potent Aβ-degrading enzyme [50, 51]. NEP dysfunction elevates endogenous Aβ levels in the mouse brain in a gene dose-dependent manner [52]. These findings suggest that NEP may have profound effects on AD pathogenesis by promoting Aβ clearance.

ACE is significantly expressed by the endothelium throughout the body and is known for regulating salt balance; this enzyme has also been shown to degrade Aβ and, more importantly, cleave Aβ42 into the less toxic Aβ40 [53, 54]. Genetic studies have revealed a link between reduced plasma ACE levels and increased AD risk [55, 56]. Furthermore, ACE overexpression by myelomonocytes leads to a reduction in brain Aβ levels [57], indicating the potential role of ACE in Aβ clearance. In addition, ECE-1, plasmin, and MMP-9 also participate in Aβ degradation [58, 59], and compelling data support a major role of MMP-9 in the degradation of Aβ compact plaques [60, 61], demonstrating its potent role in Aβ clearance.

##### Monocytes

Monocytes are a key component of the innate immune system and have multiple functions, such as the removal of debris and dead cells via phagocytosis. Multiple lines of evidence highlight the crucial role of monocytes in AD. Circulating monocytes give rise to various tissue-resident macrophages throughout the body and specialized cells, such as microglia in the brain. Peripheral monocytes are found to be able to uptake Aβ from the blood, and the phagocytosis of Aβ by monocytes is compromised in AD patients [62, 63]. These findings suggest that monocytes might play a substantial role in clearing Aβ from blood, and deficits in phagocytosis of Aβ by monocytes would contribute to the pathogenesis of AD.

Indeed, a decreased capacity of peripheral monocytes to capture Aβ resulted in increased Aβ levels [64]. In addition, the expression of monocyte cell adhesion molecules, such as ICAM-3 and P-selectin, was significantly reduced in AD patients, demonstrating that peripheral blood macrophages from AD patients displayed an impaired capacity to take up and digest Aβ [65–67]. Moreover, the expression of surface receptor TREM2, which is involved in monocyte phagocytosis, and CD33, which is involved in Aβ42 internalization, is altered in monocytes of AD patients [68, 69], suggesting that Aβ clearance by monocytes plays a substantial role in AD pathogenesis. Recent research has demonstrated that depletion of perivascular macrophages causes increased vascular Aβ levels. However, stimulation of perivascular macrophage turnover decreased the cerebral CAA load, highlighting the importance of perivascular macrophages in this AD-related disease [70]. Patrolling monocytes have been shown to infiltrate the brain and differentiate into activated macrophages in AD [71], and these circulating monocyte-derived macrophages are more efficacious than resident microglia in clearance of Aβ plaque in the brain [72]. The circulating monocyte subset could adhere to the Aβ-rich vasculature in the brain and effectively eliminate Aβ microaggregates by internalizing and transporting them from the brain vasculature to the blood [71]. Taken together, these observations outline the crucial role of the monocyte-mediated clearance of Aβ in both the brain and the periphery in AD.

##### Erythrocytes

Recent evidence suggests that Aβ is subject to erythrocyte-mediated immune adherence at every step in the pathway, where Aβ activates serum complement, and complement-opsonized Aβ peptides are captured by erythrocytes via CR1 and transported to liver and spleen for clearance [73, 74]. CR1 is deficient in erythrocytes of AD patients, and the single nucleotide polymorphisms (SNPs) associated with decreased erythrocyte CR1 increase AD risk, whereas a CR1 SNP associated with increased erythrocyte CR1 reduces AD risk [75]. These findings are helpful to establish a mechanistic link between the CR1 polymorphisms and their risks for AD [76]. Furthermore, it was recently found that Aβ antibodies can dramatically increase complement activation and opsonization of Aβ, and therefore enhance Aβ capture by human erythrocytes and macrophages [77]. These findings suggest that the peripheral mechanism cannot be ignored for the Aβ clearance by immunotherapies, and infer the potential roles of interaction between autoantibodies to Aβ and erythrocytes in the pathogenesis of AD. It is also found that the number of erythrocytes is less in AD patients than in cognitively normal control [78]. Taken together, these studies suggest that erythrocyte-mediated clearance, a major pathway for clearance of circulating pathogens, is a substantial approach for the clearance of circulating Aβ.

#### Liver-mediated Aβ clearance

When flowing into the periphery, Aβ usually binds to other molecules. Previous studies have demonstrated many transport proteins, such as albumin, ApoE, ApoJ, transthyretin (TTR), and α-2 M could bind Aβ [79–83]. However, in human plasma, the soluble form of LRP1, which sequesters 70%–90% of plasma Aβ, is the major binding protein for circulating Aβ and mediates peripheral Aβ degradation in the liver, kidneys and spleen [84].

The liver has many functions, including endocrine function, immunomodulation, lipid metabolism, and detoxification, which may all be involved in AD pathogenesis. It is proposed that once efflux from the brain occurs, Aβ is transported to the liver by high-density lipoprotein (HDL) particles [85–87], indicating the participation of the liver in peripheral Aβ clearance. Hepatocytes can act on circulating Aβ via LRP1, which is highly expressed in hepatocytes, promoting its clearance by degradation or through bile excretion [88]. Moreover, upregulating liver LRP1 expression could reverse the behavioural deficits and pathologies in the brain of APP/PS1 models [89], indicating that targeting peripheral organs, such as the liver, offers a unique therapeutic approach for Aβ clearance. In addition, the function of central circadian rhythms could influence Aβ pathogenesis in a specific manner [90]. Given that the liver is the main peripheral organ communicating with brain via the liver-brain axis, the liver might affect Aβ clearance by regulating the circadian rhythm [91]. These findings highlight the importance of the liver in Aβ clearance and the pathophysiology of AD.

#### Kidney-mediated Aβ clearance

Kidney is the main excretory organ and control levels of metabolites via regulating and filtering minerals from blood. Recent researches indicated that kidney may be involved in circulating Aβ clearance. Indeed, radiographic experiments have shown that after intracranial or intravenous infusion of I^125^-labelled Aβ, radioactivity was subsequently detected in the kidney and urine [8], besides, soluble Aβ was also detected in human urine [92]. These evidence all suggest that kidney might participate in physiological clearance of Aβ by filtering Aβ from the blood to the urine. In addition, the serum Aβ levels and brain Aβ depositions were found to be significantly increased in chronic kidney disease (CKD) patients [93, 94], indicating that the reduced kidney-mediated Aβ clearance may contribute to AD pathology in brain. Furthermore, clinical studies demonstrated the link between CKD and risk of cognitive impairment [95–97], which even independent of cerebral small-vessel disease [98], implying that Aβ accumulation caused by aberrant kidney-mediated excretion may be involved in cognitive impairment in CKD patients. These studies suggest that kidney-mediated Aβ excretion have a significant impact on removing Aβ in the brain.

#### Are intestine and skin involved in peripheral Aβ clearance?

A previous study reported the detection of Aβ deposits in the non-neural tissues of AD patients, including the skin and intestine in humans [99] and the gastrointestinal tract in animals [100]. The source of Aβ deposits in the intestine and skin is probably derived from circulating Aβ in the blood, implying that the intestine and skin may participate in peripheral Aβ metabolism.

The gastrointestinal tract is a lymphoid organ, which is heavily laden with macrophages and other immune cells. This implies that the gut has the potential capacity of clearing Aβ. Increasing data demonstrate that gut microbiota is altered in AD patients [101, 102], suggesting that the brain-gut-microbiota axis is involved in the pathogenesis of AD. It is intriguing to speculate that gastrointestine may have the function of regulating Aβ metabolism in the periphery.

Skin has been defined as an immune organ for a long time. The skin and brain, both derived from ectoderm of embryo, are physiologically and pathologically connected. Newly published data suggested that Aβ34, an Aβ species with specific length, was found in the epidermal layer in human skin [103]. The deposition of Aβ was also found in the skin of AD patients [99]. Although APP expression was detected in situ in the mammalian epidermis and predominantly in basal keratinocytes [104, 105], it remains unknown whether these Aβ deposits are from circulating blood or local cells in the skin. Our previous studies found that radiolabelled Aβ mainly accumulated in the skin after intravenous injection [8]. These findings suggest that skin may be involved in the metabolism of Aβ in the periphery. In the skin there are many macrophages which may clear local Aβ. In addition, the secretion of sweat is a potential approach to excrete Aβ. Nevertheless, whether skin functions in Aβ clearance remains largely unknown. Future studies are needed to address this topic.

### Do systemic diseases increase the AD risk via the peripheral Aβ clearance approach?

#### Disorders of systemic immunity and inflammation

Immune system abnormalities are now considered a major pathological factor in AD, and innate immunity is compromised in patients with AD. Reduced expression of Aβ phagocytic receptors and Aβ-degrading enzymes and decreased phagocytic function in mononuclear macrophages and neutrophils might impede Aβ degradation and clearance [106–108]. In regard to adaptive immunity, autoreactive antibodies related to AD pathogenesis have been studied. Specifically, elevated levels of pathogenic autoreactive antibodies and decreased levels of protective antibodies could influence Aβ clearance and deposition. In addition, compelling evidence suggests that chronic systemic inflammation, such as rheumatoid arthritis and periodontitis, promotes the AD pathogenesis [109, 110]. Proinflammatory molecules, such as TNF-α, IL-6 and IL-1β, could compromise Aβ clearance by affecting the functions of not only microglia but also peripheral monocytes and Aβ-degrading enzymes [111].

#### Hepatic dysfunction

The liver is the major organ responsible for system-wide protein synthesis and metabolic detoxification. Circulating Aβ is directly cleared by degradation in hepatocytes or indirectly cleared by regulation of the liver-mediated albumin level and Aβ-related lipid metabolism. Our previous study suggests that hepatic dysfunction, such as liver cirrhosis, is accompanied by higher levels of circulating Aβ. The reduced hepatic LRP1 levels in ageing rats contributed to decreased peripheral Aβ clearance [88, 112], suggesting that LRP1-dependent hepatocyte-mediated Aβ clearance is potentially important [113]. In addition, a recent study found that an elevated AST/ALT ratio and decreased levels of ALT were associated with AD brain biomarkers and poor cognitive performance [114], linking liver dysfunction to AD pathogenesis. Increasing evidence suggests that abnormal lipid metabolism is associated with an increased risk of AD [115], and some potential AD risk genes link to lipid metabolism [116]. Furthermore, ApoE, a liver-synthesized protein critical for AD risk, could regulate Aβ clearance via BBB transportation, enzymatic degradation and many other pathways [117].

#### Renal dysfunction

The kidney is traditionally considered to be an excretory organ, and soluble Aβ is a normal component of human urine [92]. Patients with CKD have increased circulating Aβ levels [93] and decreased cognitive functions [95, 118]. Aβ deposition is observed in the brains of CKD patients [94]. Furthermore, cerebral atrophy correlates with measures of renal function in patients with CKD [119]. These findings imply that the reduction in renal function may attenuate peripheral Aβ clearance. Therefore, it is possible that cerebral Aβ accumulation may be involved in the development of cognitive decline in CKD patients.

#### Diabetes mellitus

Numerous studies have demonstrated that patients with diabetes have an increased risk of developing AD [120]. The underlying mechanisms that link the development of diabetes with AD include the disorders of Aβ metabolism in both the brain and periphery. In patients with diabetes, excess insulin can competitively inhibit IDE-mediated Aβ degradation [49]. Moreover, diabetes also influences Aβ clearance through other mechanisms, including oxidative stress, BBB disruption, the activation of inflammatory pathways, and hypercholesterolemia [121]. In addition, insulin resistance compromises intracellular translocation of LRP1 to the plasma membrane in hepatocytes, potentially hindering hepatic clearance of circulating Aβ [112].

### Strategies for AD therapies via peripheral Aβ clearance

**Fig. 1:**
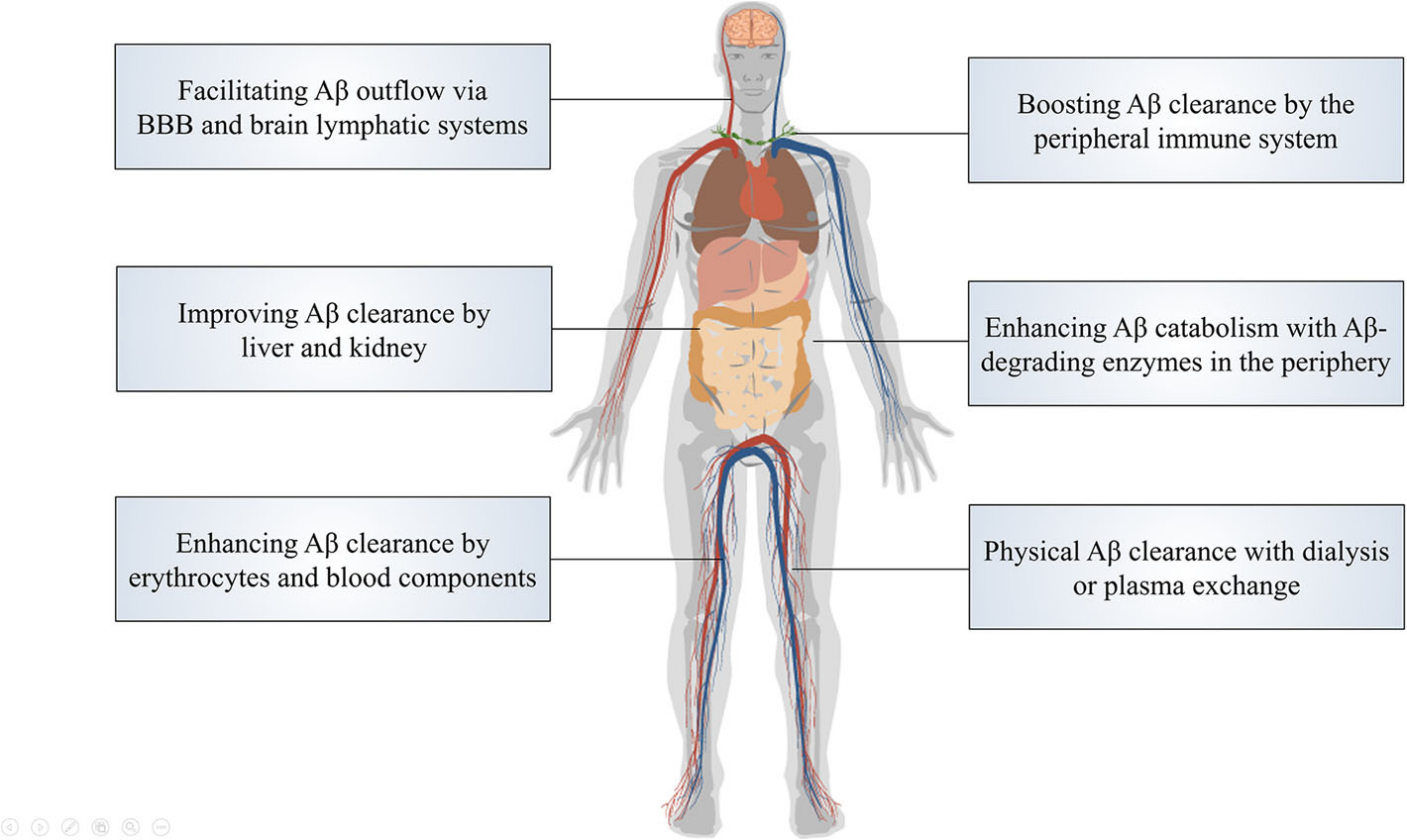
Perspectives in AD therapies via peripheral Aβ clearance. Various clearance pathways have been identified in the periphery and demonstrate potential therapeutic values. Several peripheral tissues, organs and cell types physiologically participate in Aβ clearance from the brain. Strengthening these peripheral processes is a potential approach to develop interventions for AD

Many strategies aim to alleviate AD via peripheral Aβ clearance. Here, we propose the following directions for future research (Fig. 1): (1) Maintaining the function of the BBB and brain lymphatic systems, which are critical for transporting Aβ from the brain to the periphery, could promote brain Aβ outflow and thereby attenuate Aβ accumulation in the brain. (2) Peripheral organs, such as the liver and kidney, are thought to physiologically participate in Aβ clearance. Whether liver or renal dysfunction also improves the Aβ load in the brain remains to be answered. However, it is known that strengthening liver function via herbal medicine or kidney transplant can reduce the plasma Aβ levels [94]. These findings suggest that improving the Aβ clearance capacity of the liver and kidney has therapeutic potentials. (3) Mounting evidence has demonstrated the roles of the immune system in AD pathogenesis. A cluster of AD risk gene mutations have been found to compromise the phagocytic function of Aβ by monocytes [68, 122]. In terms of adaptive immunity, antibody-based immunotherapies have been tested for AD. In addition, immunePEGliposome and antibody-functionalized polymer nanoparticle have been used to ameliorate the AD pathology in animal models [123, 124]. In these immunological modalities, the majority of antibodies locate in the blood and sequester peripheral Aβ [125]. Therefore, improving the clearance of Aβ by peripheral immunomodulation and immune cells will be a promising therapeutic strategy [126]. (4) Improving Aβ degradation by peripheral degrading enzymes is also a promising approach. Continuous expression of NEP in skeletal muscle or increased circulating NEP levels reduces the Aβ burden in AD mice [127, 128]. Additionally, peripherally derived ACE-enhanced macrophages alleviate AD pathology and behavioural defects [129]. These findings suggest that strengthening peripheral Aβ degradation is a potential AD therapeutic approach. (5) Erythrocytes and albumin enable physiological clearance of Aβ in the blood. This function can be used to develop the therapies to clear Aβ in the brain [130]. (6) Plasma albumin exchange decreases the Aβ burden in patients with AD and improves AD-related cognitive function [131]. In addition, patients who have undergone haemodialysis exhibited a reduction in Aβ deposition in the brain [94]. Furthermore, peritoneal dialysis reduces blood Aβ levels in humans and attenuates AD pathology in an APP/PS1 mouse model [132]. These observations indicated that dialysis or plasma exchange would be a potential therapeutic approach [133].

All these data suggest that increasing efflux of brain-derived Aβ and strengthening peripheral Aβ clearance can help relieve AD pathology. In addition, peripheral clearance is proposed to be a safer and easier therapeutic approach for AD than the traditional central clearance approach by introducing reagents into the brain [134]. In this regard, active removal of excess peripheral Aβ seems to be a particularly promising therapeutic strategy for AD .

However, it should be mentioned that some of the peripheral clearance pathways are not absolutely specific for Aβ. These approaches, such as Aβ-binding receptors (i.e. LRP1 and RAGE)-mediated BBB transport, enzyme (i.e. NEP and IDE)-mediated degradation, and erythrocyte-mediated immune adherence, also work for the clearance of other molecules or metabolites, implying that general enhancement of these clearance functions might cause adverse effects due to disturbance to the metabolism of other molecules. Therefore, Aβ-specific clearance methods are desirable for the development of AD therapies. Nonspecific mechanisms of Aβ clearance could also be therapeutically tractable as long as their impacts to other substrates is evaluated to be safe. In addition, the Aβ levels in the blood reflect the Aβ metabolisms in both the brain and the periphery. For example, muscle cells can also produce and release Aβ into the blood, and the Aβ clearance by liver and kidney influences the Aβ levels in the blood. This is may explain why Aβ levels in the blood do not accurately reflect the amount of Aβ levels in the brain.

## Conclusion

As there is a close interaction between Aβ metabolisms in the brain and the periphery [39], dysfunctions of Aβ metabolisms in the periphery might contribute to the development of AD, and targeting peripheral Aβ clearance represents a new opportunity for the prevention and treatment of the disease.

## Data Availability

Not applicable.

## Abbreviations

AD: Alzheimer’s disease
PD: Parkinson’s disease
ALS: Amyotrophic lateral sclerosis
CKD: Chronic kidney disease
Aβ: Amyloid-beta
NEP: Neprilysin
IDE: Insulin-degrading enzyme
MMP: Matrix metalloproteinases
ECE: Endothelin-converting enzyme
ACE: Angiotensin converting enzyme
BBB: Blood-brain barrier
LRP-1: Lipoprotein receptor-related protein 1
α2M: Alpha-2-microglobulin
PICALM: Phosphatidylinositol-binding clathrin assembly protein
Pgp: P-glycoprotein
RAGE: Advanced glycation end products
CAA: Cerebral amyloid angiopathy
VRS: Virchow-Robin space
VEGFC: Vascular endothelial growth factor C
CR1: Complement receptor 1
GWAS: Genome-wide association studies
TTR: Transthyretin
